# Wrist-wearable bioelectrical impedance analyzer with miniature electrodes for daily obesity management

**DOI:** 10.1038/s41598-020-79667-3

**Published:** 2021-01-13

**Authors:** Myoung Hoon Jung, Kak Namkoong, Yeolho Lee, Young Jun Koh, Kunsun Eom, Hyeongseok Jang, Wonjong Jung, Jungmok Bae, Jongae Park

**Affiliations:** 1 Healthcare Sensor Lab, Device Research Center, Samsung Advanced Institute of Technology, Samsung Electronics Co., Ltd., 130 Samsung-ro, Yeongtong-gu, Suwon-si, 443-803 Gyeonggi-do Korea; 2 GI Innovation, Inc., A-1116 Tera Tower, 167, Songpa-daero, Songpa-gu, Seoul, Korea; 3 Samsung Strategy and Innovation Center, Samsung, Inc., 3655 North 1st Street, San Jose, CA 95134 USA

**Keywords:** Health care, Disease prevention, Weight management

## Abstract

Bioelectrical impedance analysis (BIA) is used to analyze human body composition by applying a small alternating current through the body and measuring the impedance. The smaller the electrode of a BIA device, the larger the impedance measurement error due to the contact resistance between the electrode and human skin. Therefore, most commercial BIA devices utilize electrodes that are large enough (i.e., 4 × 1400 mm^2^) to counteract the contact resistance effect. We propose a novel method of compensating for contact resistance by performing 4-point and 2-point measurements alternately such that body impedance can be accurately estimated even with considerably smaller electrodes (outer electrodes: 68 mm^2^; inner electrodes: 128 mm^2^). Additionally, we report the use of a wrist-wearable BIA device with single-finger contact measurement and clinical test results from 203 participants at Seoul St. Mary’s Hospital. The correlation coefficient and standard error of estimate of percentage body fat were 0.899 and 3.76%, respectively, in comparison with dual-energy X-ray absorptiometry. This result exceeds the performance level of the commercial upper-body portable body fat analyzer (Omron HBF-306). With a measurement time of 7 s, this sensor technology is expected to provide a new possibility of a wearable bioelectrical impedance analyzer, toward obesity management.

## Introduction

Consumer interests in personalized health, including fitness and weight management, have been increasing. Body composition measurements are known to be useful in managing total body energy balance, and some of the techniques used include tracer dilution, densitometry, dual-energy X-ray absorptiometry (DEXA), air displacement plethysmography, and bioelectrical impedance analysis (BIA). Among them, BIA has recently attracted attention as a simple and non-invasive modality.

BIA is a commonly used method for estimating body fat by measuring the electrical impedance of a human body. The percentage body fat is calculated by inputting this body impedance value into a predetermined regression equation from an appropriately chosen population data^1–7^. The parameters used in the regression equation are generally age, gender, height, weight, and impedance.

It is well accepted that upper body (hand-to-hand) BIA is useful for estimation of visceral and abdominal fat, while lower body (leg-to-leg) BIA is useful for estimation of subcutaneous fat. Lower body BIA device is usually scale-type and is convenient because weight information is automatically acquired during measurements. However, it also has a size limitation. It is considered that the upper body BIA device is more suitable for small form-factor wearable devices such as a wristwatch^8^.

However, there always exists some contact resistance between the electrode and the human skin so that the measured impedance has a different value from the actual one, and this causes some errors in estimation of percentage body fat^9,10^. In order to solve this problem, commercial BIA body fat analyzers usually take advantage of a 4-point measurement that is known to reduce the effect of contact resistance. However, even with the 4-point measurement, there also exists an impedance error when the input impedance of the voltage-measuring part and the output impedance of the current source are not much larger than the contact resistance. For this reason, most commercial BIA body fat analyzers adopt electrodes that are large enough to counteract the effect of contact resistance, i.e., 4 × 1400 mm^2^ (Omron HBF-306) electrodes. However, electrodes with such a large size cannot fit into a small form-factor wearable device, such as a wristwatch.

Recently, studies on wearable BIA device have been conducted, including studies on wearable BIA devices^11,12^, cuff-less blood pressure sensors^13,14^, and bioelectrical impedance spectroscopy^15^. For the wearable solutions, accurate measurement of impedance with miniature electrodes is becoming more important.

Herein, we report a novel wrist-wearable bioelectrical impedance analyzer that can compensate for not only the contact resistance of current-applying electrodes but also the voltage-sensing electrodes, such that body impedance can be accurately estimated even with considerably small sizes of electrodes (outer electrodes: 68 mm^2^; inner electrodes: 128 mm^2^).

## Methods

### Wrist-wearable bioelectrical impedance analyzer using single finger

**Figure 1:**
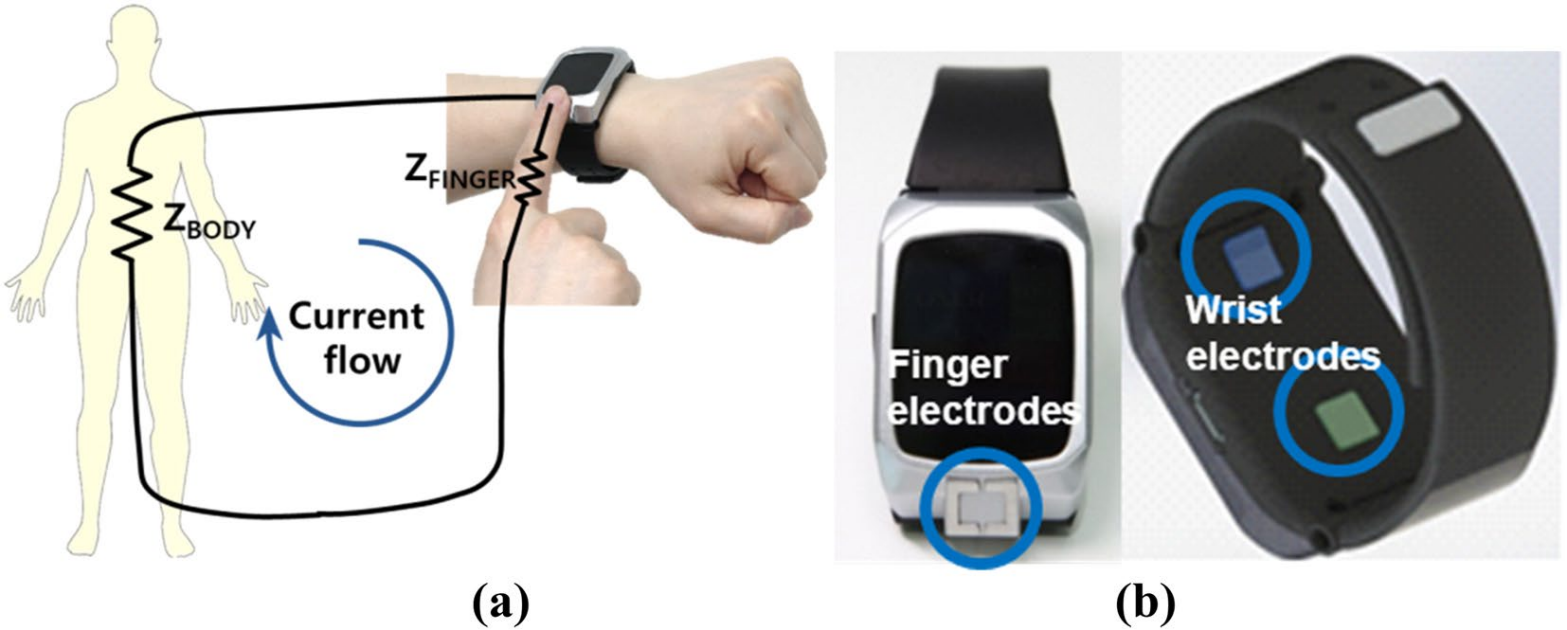
Bioelectrical impedance measurement using (**a**) single-finger and (**b**) watch-type BIA devices. The positions of 4 electrodes are shown (one pair for the finger on the top side and the other pair for the wrist on the bottom side of the main body of the device).

We developed a wristwatch-type bioelectrical impedance analyzer that provides users with convenient measurement experience by using only one finger, i.e. the index finger. Two pairs of electrodes were installed on the main body of the watch-type device: one pair (current electrode: 64 mm^2^; voltage electrode: 64 mm^2^) was positioned on the bottom of the main body of the device for contact with the wrist, and the other pair (current electrode: 34 mm^2^; voltage electrode: 64 mm^2^) was positioned on top for contact with the index finger as shown in Fig. 1.

The electrodes of our device are quite small compared to those of a traditional device such as the portable upper-body type device (Omron HBF-306): 196 mm^2^ (present device) vs. 5600 mm^2^ (Omron HBF-306). As the size of electrodes decreases, contact resistance increases and therefore needs to be compensated for in an appropriate way.

### Contact resistance

Contact resistance has been a big conundrum in bioelectrical research. Contact resistance refers to electrical impedance and depends on the electrode area and the resistivity at the electrode-to-skin interface. There are two configurations of electrodes for measuring bio-impedance: one is two-electrode method, and the other is four-electrode method. Two-electrode method uses single pair of electrodes to apply a current and measure the voltage drop along them. This method was proposed by Thomasset^16^ who conducted the original studies with electrical impedance measurement in total body water estimation, using needle-type electrodes in 1963. This method has the advantage of simple circuit and system structure due to the small number of electrodes involved, but it suffers from low accuracy due to contact resistance. Four-electrode method was developed by Hoffer et al*.*^17^ and Nyboer^18^ to reduce measurement error due to contact resistance. This method uses two pairs of electrodes and separates current-applying electrodes from voltage-measuring electrodes. Two current electrodes drive electricity into a human body, and two voltage electrodes detect the voltage drop along the human body. An ideal voltmeter should have an input impedance of infinity, and there should be no current flow on the signal path of voltage electrodes so that voltage drop can be measured accurately.

In order to study the impact of electrode size and state of the skin and electrode, contact resistance and body impedance were measured with several electrode sizes (10 × 8 mm^2^, 8 × 5 mm^2^, and 5 × 4 mm^2^) and for two skin surface states (without conductive gel and with conductive gel). The conductive gel fills the gaps between the skin and the electrode, thus reducing skin contact resistance effectively. The measurement was conducted using a prototype bioelectrical impedance measurement system with four-electrode method.

**Figure 2:**
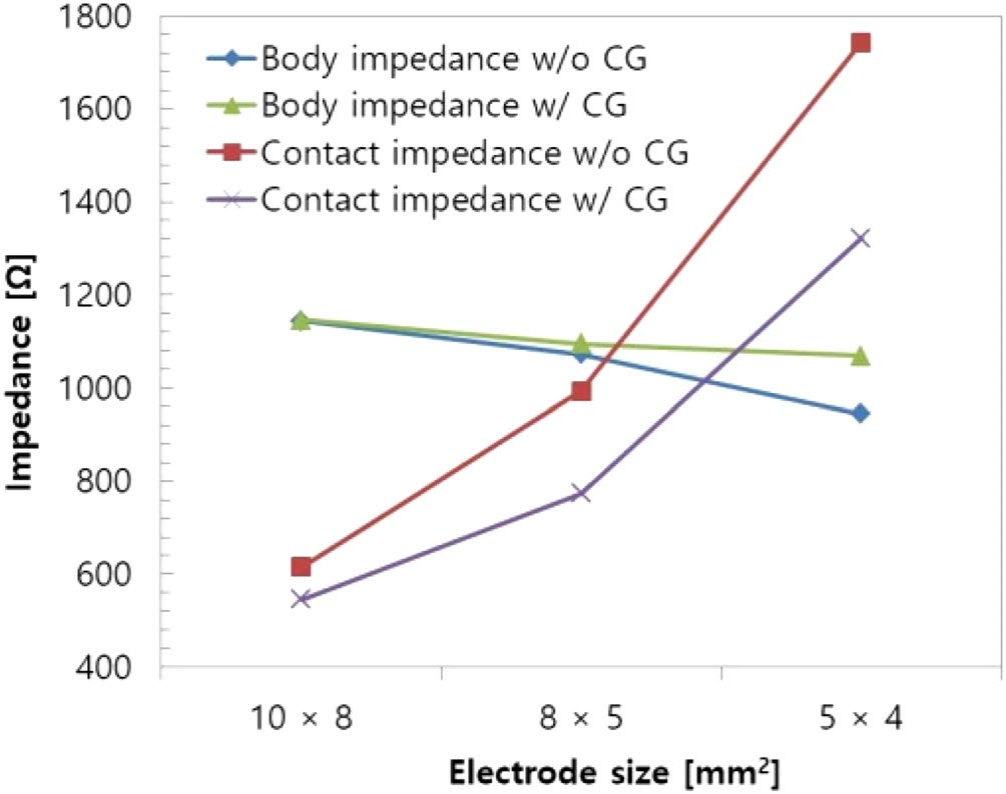
Contact resistance vs. electrode area and state of skin–electrode contact interface (*CG* conductive gel, *w/o* without, *w/* with).

As shown in Fig. 2, contact resistance increases as electrode size decreases and skin dryness increases. As a result, the measured impedance has a different value from the actual one and propagates into the estimated percentage body fat value. In order to solve this problem, most commercial devices adopt large electrodes. However, for a wearable device, the small size of electrodes is essential, so more effective solutions for accurately measuring body impedance with small electrodes are required.

### Contact resistance compensation

**Figure 3:**
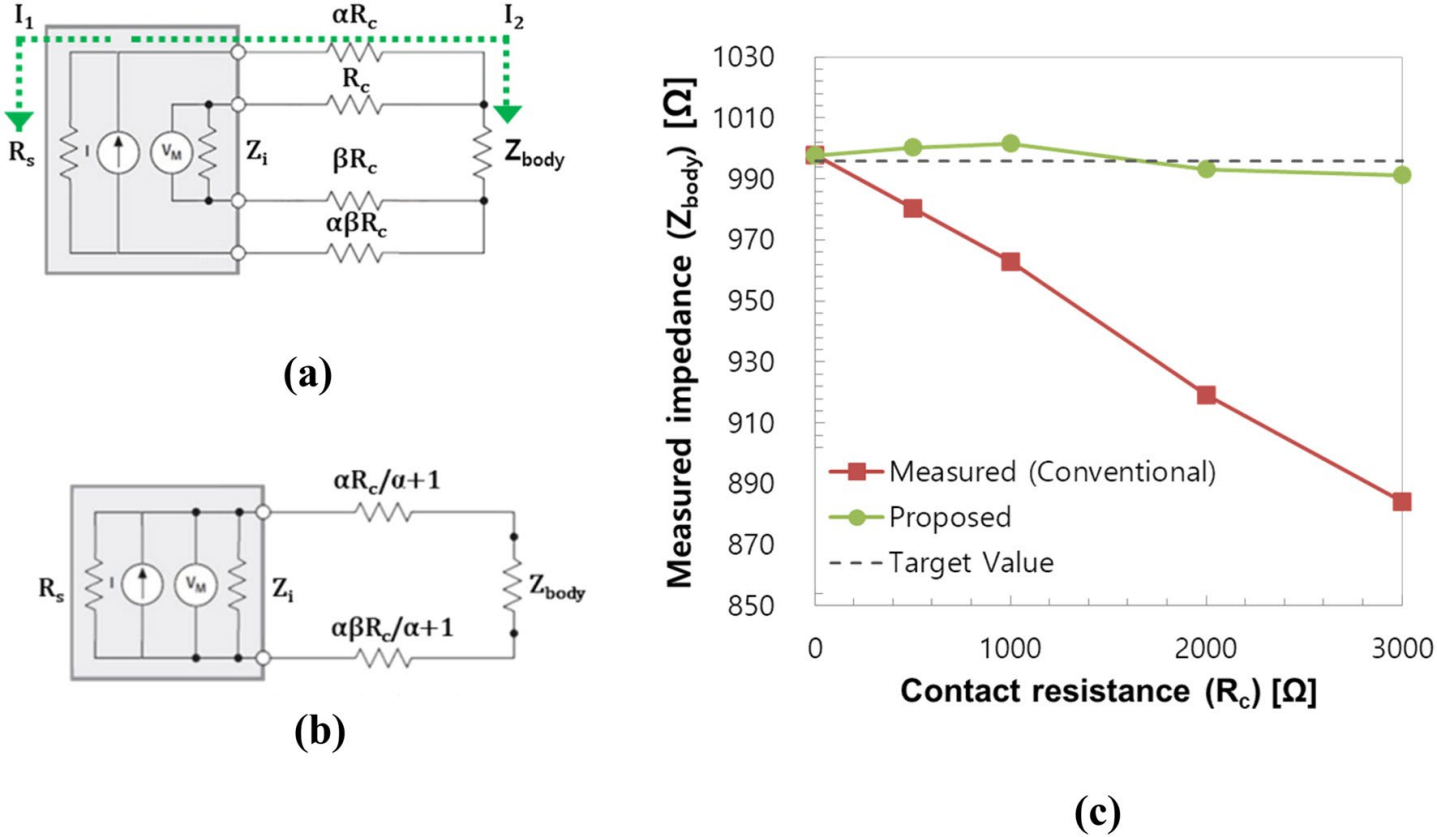
Contact resistance compensation method. (**a**) 4-point measurement mode, (**b**) 2-point measurement mode, and (**c**) contact resistance compensation effect. (|*Z*_*body*_|= 1 kΩ, |*Z*_*i*_|= 2 MΩ, *R*_*s*_ = 50 kΩ). *Z*_*body*_ measured impedance, *Z*_*i*_ input impedance of the voltmeter, *R*_*s*_ output impedance of the current source, *R*_c_ contact resistance, *I* current, *V*_*M*_ measured voltage, *α* the size ratio of voltage to current electrode, *β* the size ratio of finger to wrist electrode.

We propose a contact resistance compensation method for accurate body impedance measurement even with small electrodes, and which is independent of contact resistance. The measurement can be divided into two stages. Figure 3a,b show the block diagram of analog front-end (AFE) with contact resistance compensation function. In the 4-point measurement mode, voltage drop on the two voltage electrodes was measured while applying electrical current through the two current electrodes. Assuming the size ratio of voltage to current electrode as α, and the size ratio of finger to wrist electrode as β, in Fig. 3a, load impedance detected by the current source can be expressed by Eq. (1):

1 $$R_{L} = \alpha \left( {\beta + 1} \right)R_{c} + \frac{1}{{\frac{1}{{\left| {Z_{body} } \right|}} + \frac{1}{{\left( {\beta + 1} \right)R_{c} + \left| {Z_{i} } \right|}}}}$$

*|Z*_*body*_| is the measured impedance; *|Z*_*i*_| is the input impedance of the voltmeter, and *R*_c_ is the contact resistance.

The electric current of the current source (*I*_*s*_) is divided into two parallel branches, namely internal resistance (*I*_*1*_) loop and external loop (*I*_*2*_). The current through the external current loop is calculated as Eq. (2):

2 $$I_{2} = I_{s} \times \frac{{R_{s} }}{{R_{s} + R_{L} }} = { }I_{s} \times \frac{{R_{s} }}{{R_{s} + \alpha \left( {\beta + 1} \right)R_{c} + \frac{1}{{\frac{1}{{\left| {Z_{body} } \right|}} + \frac{1}{{\left( {\beta + 1} \right)R_{c} + \left| {Z_{i} } \right|}}}}}}$$

For a finite |*Z*_*i,*_|, this current (*I*_*2*_) is also divided into |*Z*_*body*_| and |*Z*_*i*_|. The voltage meter measures the voltage drop of |*Z*_*i*_|, which can be expressed as Eq. (3):

3 $$\begin{aligned} V_{M} & = \left| {Z_{i} } \right| \times \left( {I_{2} \times \frac{{\left| {Z_{body} } \right|}}{{\left( {\beta + 1} \right)R_{c} + \left| {Z_{i} } \right| + \left| {Z_{body} } \right|}}} \right) \\ & = { }I_{s} { } \times { }\left| {Z_{i} } \right| \times \frac{{\left| {Z_{body} } \right|}}{{\left( {\beta + 1} \right)R_{c} + \left| {Z_{i} } \right| + \left| {Z_{body} } \right|}} \times \frac{{R_{s} }}{{R_{s} + \alpha \left( {\beta + 1} \right)R_{c} + \frac{1}{{\frac{1}{{\left| {Z_{body} } \right|}} + \frac{1}{{\left( {\beta + 1} \right)R_{c} + \left| {Z_{i} } \right|}}}}}}{ } \\ \end{aligned}$$

Since the measured voltage (*V*_*m*_) and source current (*I*_*s*_) are known, impedance in the 4-point measurement mode can be represented as Eq. (4):

4 $$\left| {Z_{4p} } \right| = \frac{{V_{M} }}{{I_{s} }} = \left| {Z_{body} } \right| \times \left[ {\frac{1}{{1 + \frac{{\left| {Z_{body} } \right| + \left( {\beta + 1} \right)R_{c} }}{{\left| {Z_{i} } \right|}}}}} \right] \times \left[ {\frac{{R_{s} }}{{R_{s} + \alpha \left( {\beta + 1} \right)R_{c} + \frac{1}{{\frac{1}{{\left| {Z_{body} } \right|}} + \frac{1}{{\left( {\beta + 1} \right)R_{c} + \left| {Z_{i} } \right|}}}}}}} \right]$$

where |*Z*_*4p*_| is the measured 4-point impedance, |*Z*_*body*_| is the body impedance to be determined, |*Z*_*i*_| is the input impedance of the voltmeter, *R*_*s*_ is the output impedance of the current source, and *R*_*c*_ is the contact resistance, also to be determined. |*Z*_*i*_| and *R*_*s*_ are known values from instrument providers. The first square bracket in Eq. (4) shows the effect of contact resistance at the voltage electrodes. When the voltmeter has a finite input impedance (|*Z*_*i*_|), the measured 4-point impedance decreases with increase in contact resistance due to the voltage drop at the voltage electrodes. The second square bracket in Eq. (4) shows the effect of contact resistance at the current electrodes. When the current source has a finite output impedance (*R*_*s*_), the measured 4-point impedance also decreases with the increase in contact resistance due to the decrease in current flow into the human body.

In the 2-point measurement mode, voltage and current electrodes on the same side are electrically connected with internal analog switches, so that four electrodes can be operated as two electrodes. The measured 2-point impedance (|*Z*_*2*_* |*) can be expressed by Eq. (5):

5 $$\left| {Z_{2p} } \right| = \frac{1}{{\frac{1}{{\left| {Z_{body} } \right| + \frac{{\alpha \left( {\beta + 1} \right)}}{\alpha + 1}R_{c} }} + \frac{1}{{\left| {Z_{i} } \right|}} + \frac{1}{{R_{s} }}}}$$

Because there are two equations (Eq. (4) and (5)) and two unknown quantities (|*Z*_*body*_| and *R*_*c*_), body impedance can be determined independent of contact resistance as Eq. (6):

6 $$\left| {Z_{body} } \right| = \left| {Z_{4p} } \right|\frac{{\left( {\frac{\alpha + 1}{{2\alpha }}\theta + \left| {Z_{i} } \right|} \right)\left( {\frac{\alpha + 1}{2}\theta + R_{s} } \right)}}{{\left| {Z_{4p} } \right|\left( {\frac{{\left( {\alpha + 1} \right)^{2} }}{2\alpha }\theta + \alpha \left| {Z_{i} } \right| + \frac{{R_{s} }}{\alpha }} \right) + \left| {Z_{i} } \right|R_{s} }} \theta = \frac{2}{{\frac{1}{{\left| {Z_{2p} } \right|}} - \frac{1}{{\left| {Z_{i} } \right|}} - \frac{1}{{R_{s} }}}}$$

We performed an experiment to verify the proposed method by using a simple electrical circuit. Discrete resistors were used to model body impedance and contact resistance. The resistance value of the model for body impedance (|*Z*_*body*_|) was fixed at 1000 Ω.

Figure 3c shows the variation of measured impedance values before and after contact resistance compensation when the contact resistance varied from 0 to 3 k Ω. The maximum measurement error after contact resistance compensation was reduced to about − 0.5%, whereas the conventional 4-point measurement had an error as large as − 11.2%.

### Hardware setup

**Figure 4:**
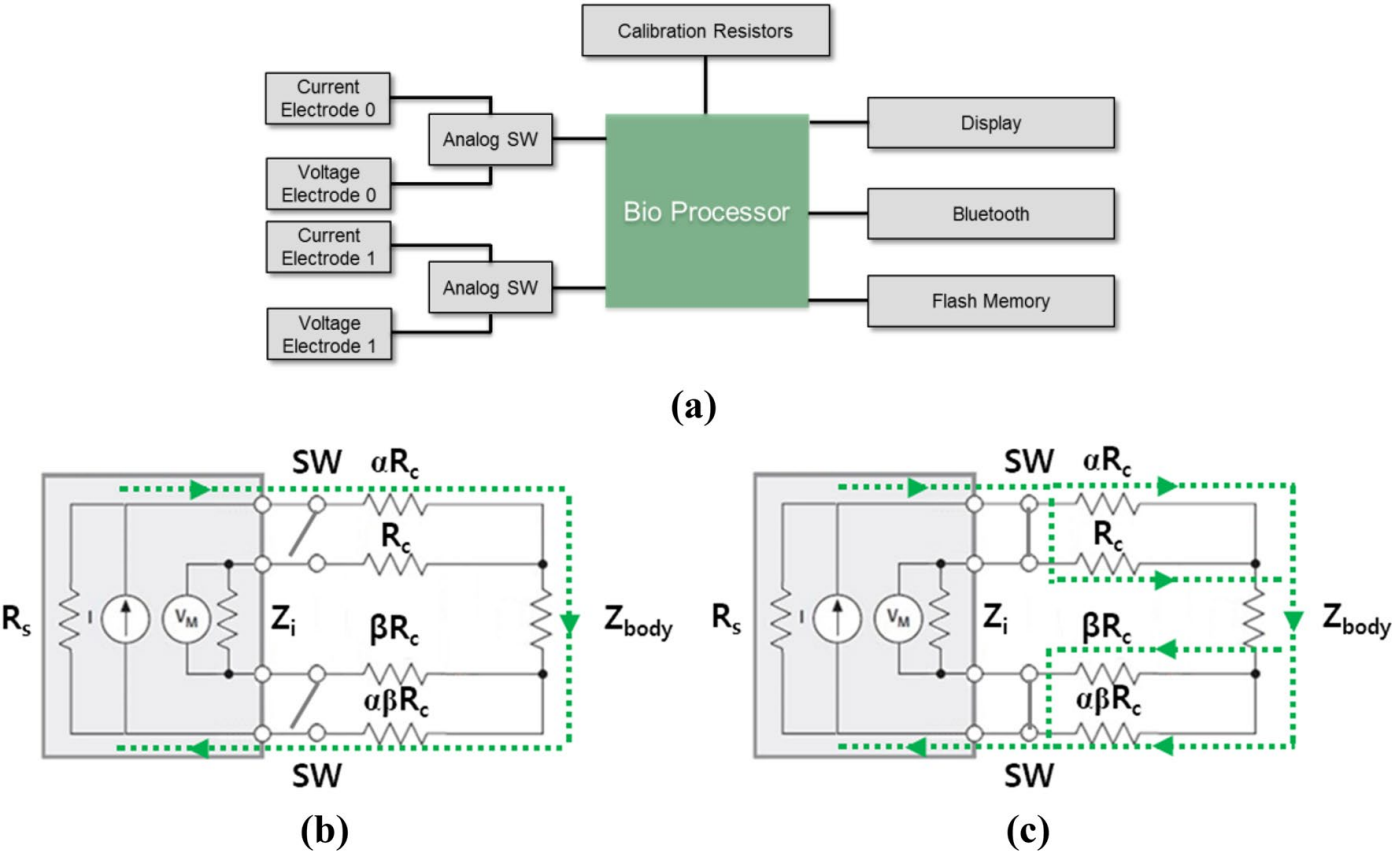
(**a**) Hardware block diagram of the overall system, (**b**) current path for 4-point measurement mode, and (**c**) current path for 2-point measurement mode. *Z*_*body*_ measured impedance, *Z*_*i*_ input impedance of the voltmeter, *R*_*s*_ output impedance of the current source, *R*_c_ contact resistance, *I* current, *V*_*M*_ measured voltage, SW switch, *α* the size ratio of voltage to current electrode, *β* the size ratio of finger to wrist electrode.

A novel wrist-wearable bioelectrical impedance analyzer with contact resistance compensation function was developed. Figure 4a shows the block diagram of the developed BIA device.

The electrodes part is composed of two current-driving electrodes and two voltage-sensing electrodes. The total area of finger electrodes (a pair of one current electrode and one voltage electrode on the top side of the device) was 68 mm^2^ and that of wrist electrodes (another pair of one current electrode and one voltage electrode on the bottom side of the device) was 128 mm^2^. The AFE (S3FBP5A, BioProcessor2, Samsung Electronics) delivers 30 μA sinusoidal alternating current with 50 kHz frequency to the two current electrodes and measures voltage drop between the two voltage electrodes. Acquired voltage values were converted to digital signal by analog-to-digital converter (ADC), and this digital code was converted to impedance value with a calibration curve which had been made by calibration process. The internal micro controller unit of BioProcessor2 calculated body fat, lean body mass, and body water volume using impedance data and user profile information such as height, age, weight, and gender. The measured data was displayed on liquid crystal display. Bluetooth was used for data transfer between body fat analyzer and a personal computer, and external flash memory was used for user data storage.

Contact resistance compensation function was adapted to our bioelectrical impedance analyzer. The contact resistance compensation circuit included two analog switches. One analog switch was connected between the current and voltage path of the finger electrodes, and the other was connected between the current and voltage path of the wrist electrodes. For the 4-point measurement mode, analog switches were turned off, and for the 2-point measurement mode, analog switches were turned on for electrical connection of each voltage and current electrode pair. This very simple and small compensation circuit had a flexibility that allowed easy adaptation to variable AFEs.

The dynamic range (the range of measureable impedance) was configured to cover the range of body impedance and contact resistance. TX dynamic range (the range of impedance that current source can drive) and RX dynamic range (the range of impedance that voltmeter can measure) should satisfy the overall system dynamic range required. Figure 4b,c show the current paths for the 4-point and 2-point measurement modes, respectively. In the 4-point measurement mode, current source should have a dynamic range of *2R*_*c*_ +|*Z*_*body*_| because the current flows into the human body through two series contact resistance, and the voltmeter should have a dynamic range of |*Z*_*body*_| because it monitors voltage drop on the body. In the 2-point measurement mode, the impedance that the current source drives and the impedance that the voltmeter measures are the same as *R*_*c*_ +|*Z*_*body*_|. Since the dynamic range of current source and voltmeter should satisfy each condition of the measurement mode, TX dynamic range should cover 0 to *2R*_*c*_ +|*Z*_*body*_|, and RX dynamic range should cover 0 to *R*_*c*_ +|*Z*_*body*_|. Based on our user data from 148 volunteers in 2014, TX and RX dynamic range were set as 15 kΩ and 10 kΩ, respectively, by adjusting the driving current level^10^.

To improve measurement accuracy along the wide dynamic range stated above, a calibration algorithm that adopts 4-point coordinate conversion is proposed, in which four high-precision reference resistors are used to reduce the errors in three resistance sections. The ADC output code was converted to impedance by calibration process. The ADC output code and body impedance (|*Z*_*body*_|) have a nonlinear relationship due to the finite input impedance of the voltmeter (|*Z*_*i*_|) and the finite output impedance of the current source (*R*_*s*_), since the equivalent impedance detected by the voltmeter is the parallel combination impedance of body impedance (|*Z*_*body*_|), |*Z*_*i*_|, and *R*_*s*_ as in Eq. (7). (Note that contact resistance (*R*_*c*_) is zero during the calibration process).

7 $$\left| {Z_{READ} } \right| = \left| {Z_{body} } \right|//\left| {Z_{i} } \right|//R_{s} = \frac{{\left| {Z_{body} } \right|\left| {Z_{i} } \right|R_{s} }}{{\left| {Z_{body} } \right| + \left| {Z_{i} } \right| + R_{s} }}$$

**Figure 5:**
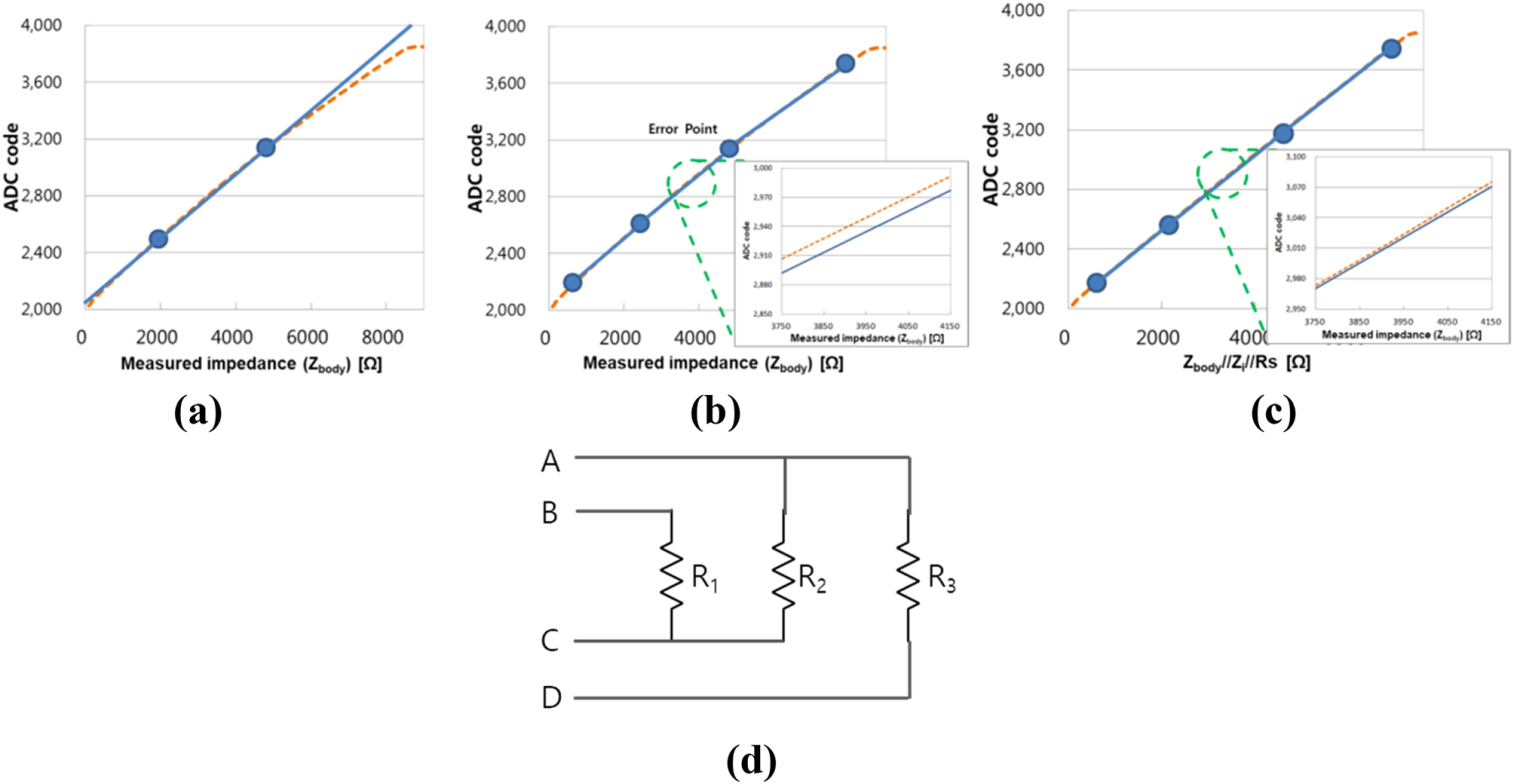
Calibration curve and measurement error at midpoint between two reference points for (**a**) 2-point calibration, (**b**) 4-point calibration, and (**c**) 4-point coordinate conversion calibration. (**d**) Calibration circuit for 4 reference resistance values (A‒C route: R_2_, A‒D route: R_3_, B‒C route: R_1_, B‒D route: R_1_ + R_2_ + R_3_). *ADC* analog-to-digital converter, *Z*_*i*_ input impedance of the voltmeter, *R*_*s*_ output impedance of the current source.

In the calibration curve, the measurement on the x-axis is changed from reference impedance to parallel combination impedance of reference impedance, |*Z*_*i*_|, and *R*_*s*_. This change enhances the linearity of the calibration curve and the accuracy of measurement. Figure 5a‒c show the measurement error for conventional 2-point calibration, 4-point calibration, and the proposed 4-point coordinate conversion calibration. The dashed lines are ideal calibration curves, and the solid lines are extracted calibration curves derived by calibration process. It is easily seen from Fig. 5a‒c that 4-point coordinate conversion calibration minimizes calibration error, compared with other conventional methods. Calibration algorithm was developed using C code and loaded as firmware of the device. Whenever the device is turned on, self-calibration is conducted using 4 reference resistance values as shown in Fig. 5d.

**Figure 6:**
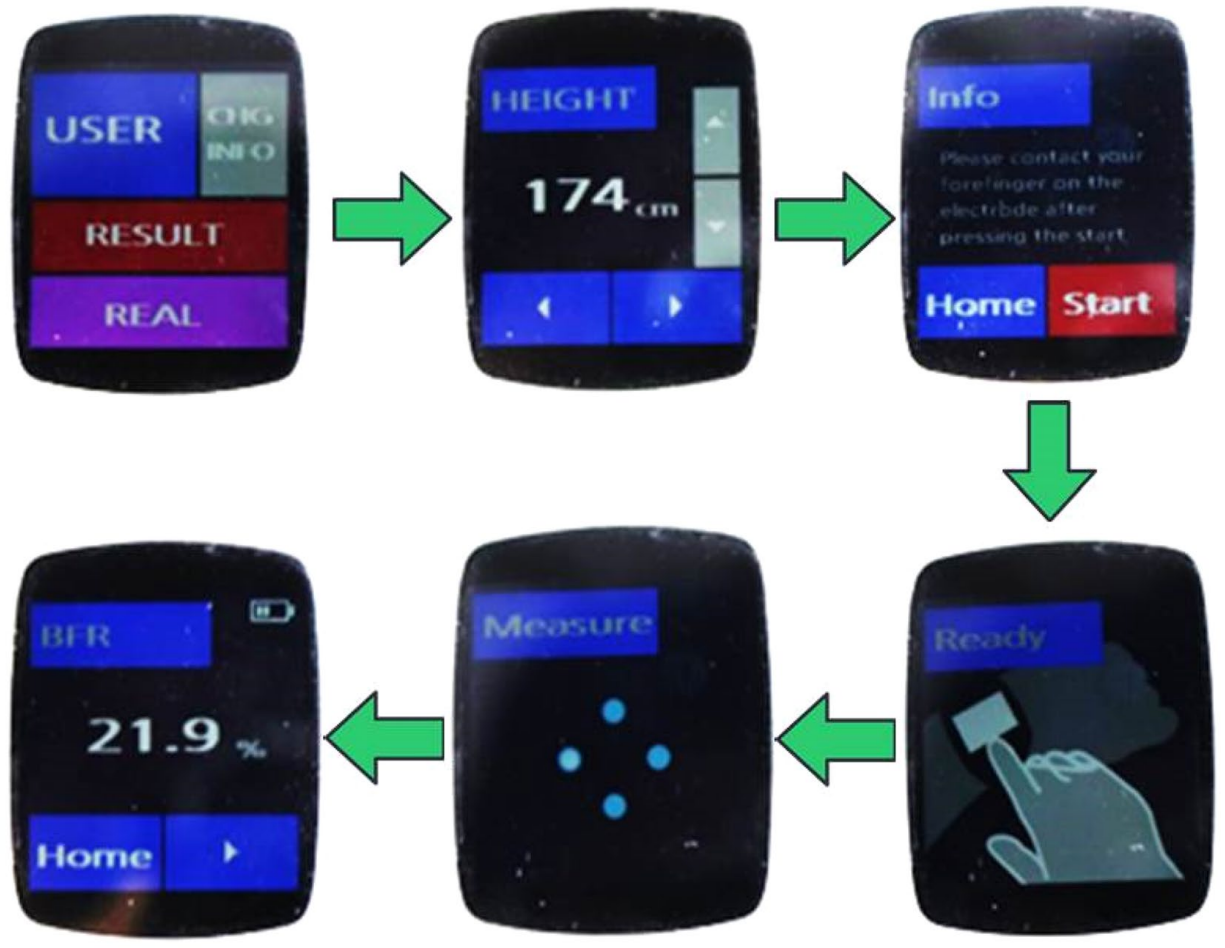
Measurement procedure and corresponding graphical user interface of the wrist-wearable bioelectrical impedance analyzer.

Figure 6 shows the measurement procedure and corresponding graphical user interface of our wrist-wearable device. On the home screen, a user can register information (gender, age, height, and weight) by touching the [CHG INFO] icon. If the user is already registered on the device, registration process can be skipped by touching [USER] icon. The measurement is initiated by touching the [START] icon. When the proper posture is maintained, BIA measurement begins automatically. It takes about 7 s to complete the test: 3 s for 4-point measurement, 1 s for measurement mode change, and 3 s for 2-point measurement. When the measurement is completed, percentage body fat, lean mass, and basal metabolic rate are shown on the screen.

### Clinical test

To evaluate the accuracy of our bioelectrical impedance analyzer, a clinical test was conducted on 203 volunteers who were recruited at Seoul St. Mary’s Hospital. The study population consisted of 18‒68-year-old healthy male (n = 101) and female (n = 102) volunteers. Participants were recruited to have as uniform distributions as possible on the bases of gender, age, and body mass index (BMI). Ages were divided into 6 groups (18 and 19, 20‒29, 30‒39, 40‒49, 50‒59, and 60‒69 years), and weights were divided into 3 ranges, which are underweight (BMI < 18.50 kg/m^2^), normal (18.50 ≤ BMI ≤ 24.99 kg/m^2^), and overweight (BMI > 25.0 kg/m^2^)^19^. Participants’ characteristics are shown in Table 1. The BIA pretesting client guidelines^20^ in Table 2 were explained to all volunteers before the clinical test.

**Table 1:** Physical characteristics of the subjects.

Variables	Men (n = 101)		Women (n = 102)	
	Mean (s.d.)	Min‒max	Mean (s.d.)	Min‒max
Age (years)	38.0 (15.4)	18‒66	38.5 (15.9)	18‒68
Height (cm)	172.1 (6.0)	155‒192	160.4 (5.7)	148‒177
BMI (kg/m^2^)	24.0 (3.3)	18‒34	22.2 (3.5)	16‒31
Weight (kg)	71.3 (11.0)	53‒104	57.1 (8.8)	42‒83

*s.d.* standard deviation, *BMI* body mass index.

**Table 2:** Bioelectrical impedance analysis client pretesting guidelines^20^.

1	No eating or drinking within 4 h of the test
2	No exercise within 12 h of the test
3	Client should urinate within 30 min of the test
4	No alcohol consumption within 48 h of the test
5	No diuretic medications within 7 days of the test
6	No testing of female clients who perceive they are retaining water during that stage of their menstrual cycle

Four different devices were used in the clinical test: our wrist-wearable device, a whole-body composition analyzer (InBody 720), an upper-body portable body fat analyzer (Omron HBF-306), and a DEXA instrument (GE Lunar Prodigy). The study was approved by the Institutional Review Board of Seoul St. Mary’s Hospital (KC15DISI0610), and all experiments were performed in accordance with relevant guidelines and regulations of the Medical Ethics Committee of Seoul St. Mary’s Hospital. For the approval of the review board, our bioelectrical impedance analyzer was registered as a broadcasting and communication equipment (MSIP-REM-SEC-SAIT-MyLean100) by the Ministry of Science, ICT and Future Planning (MSIP), Republic of Korea).

Written informed consent was obtained from each volunteer before the clinical test. To undergo the test, participants changed into a light gown in order to control the weight of clothes. All metal items were removed from the participants to ensure accuracy of measurement. Then anthropometric measurement was conducted by a skilled nurse. After anthropometric measurement, body impedance and body composition data were measured using the whole-body composition analyzer and the upper-body portable body fat analyzer. Next, the DEXA instrument was used to measure the reference body composition. Finally, our wrist-wearable device was used to measure body impedance.

Statistical analysis was performed after data acquisition. Bioelectrical impedance equation was derived for our wrist-wearable device: multiple linear regression with five independent variables (height, age, gender, weight, and height^2^/impedance) and one dependent variable (percentage body fat or lean body mass) was conducted using DEXA as a reference instrument. The accuracy of each device was compared to that of others.

## Results and discussion

**Figure 7:**
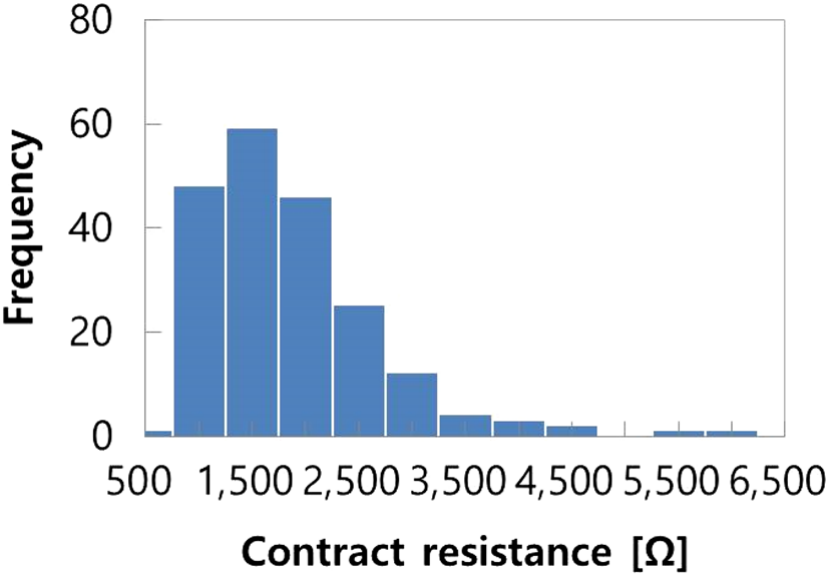
Calculated contact resistance distribution among participants in the clinical test.

Our study explored a novel method that uses considerably small electrodes that can be adapted into small devices, such as a wristwatch. Figure 7 shows the calculated contact resistance distribution of the study participants. While the average value was 1808 Ω, it is notable that the maximum value was as high as 6301 Ω.

**Figure 8:**
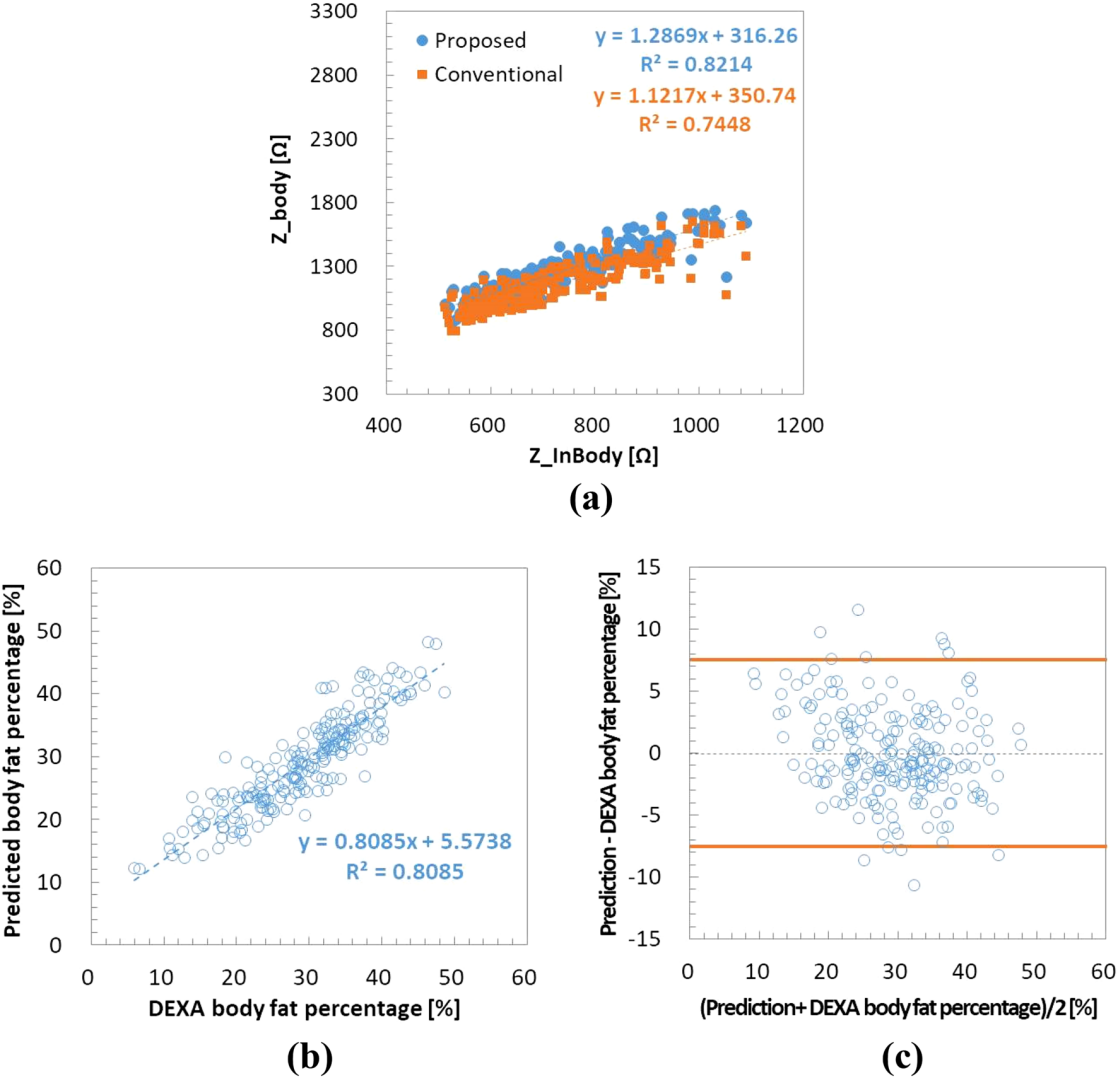
(**a**) Impedance correlation with contact resistance compensation (blue dots) and without contact resistance compensation (orange dots). Body fat algorithm accuracy (n = 203) assessment with (**b**) correlation plot and (**c**) Bland–Altman plot between our wrist-wearable bioelectrical impedance analyzer and the reference instrument (DEXA). *DEXA* dual-energy X-ray absorptiometry.

Figure 8a shows the impedance correlation between our device and the whole-body composition analyzer. The coefficient of determination (R^2^) of impedance was 0.7448 (correlation coefficient, R = 0.863) for the traditional 4-point measurements method while R^2^ after contact resistance compensation was 0.8214 (R = 0.906). This result shows that there is a strong correlation for impedance measurements between the wrist-wearable bioelectrical impedance analyzer and the whole-body bioelectrical impedance analyzer, and the proposed contact resistance compensation method improves the correlation coefficient effectively.

Figure 8b shows the correlation of percentage body fat measurement between our wrist-wearable bioelectrical impedance analyzer and the reference instrument (DEXA), from which it can be seen that R is 0.899 (R^2^ = 0.8085). Figure 8c shows the Bland–Altman plot of percentage body fat between our wrist-wearable bioelectrical impedance analyzer and the reference instrument^21^, where the orange lines indicate the range of standard error of estimate (SEE) between ‒2SEE and + 2SEE. The SEE was estimated to be 3.8 body fat percentage (%BF). It can be seen that the errors between the two instruments are randomly distributed without any skewed tendency and 94.1% of errors are located within ± 2SEE limits.

Table 3 shows the comparison of accuracy in measurement of percentage body fat by the whole-body composition analyzer, the upper-body portable body fat analyzer, and our wrist-wearable bioelectrical impedance analyzer. It is notable that our wrist-wearable device (R = 0.899, SEE = 3.8%BF) produced more accurate results than the commercial upper-body portable body fat analyzer (R = 0.893, SEE = 4.7%BF), more so with quite a smaller size of electrodes.

**Table 3:** Comparison of correlation coefficient and standard errors of estimate (SEE) of percentage body fat (%BF) among the whole-body composition analyzer (InBody 720), the upper-body portable body fat analyzer (Omron HBF-306), and our wrist-wearable bioelectrical impedance analyzer.

	InBody 720	Omron HBF-306	Present study
R	0.946	0.893	0.899
SEE (%BF)	3.7	4.7	3.8
Measurement Time (s)	150	7	7
Device type	Stationary	Handheld	Wrist-wearable

## Conclusions

We developed a novel wrist-wearable bioelectrical impedance analyzer with a contact resistance compensation function such that bioelectrical impedance can be accurately estimated even with considerably small sizes of electrodes (outer electrodes: 68 mm^2^; inner electrodes: 128 mm^2^). The correlation coefficient and the SEE of percentage body fat relative to the DEXA instrument were estimated to be 0.899 and 3.8%BF, respectively, which are above the level of performance of the commercial upper-body portable body fat analyzer. Considering that the measurement time of our wrist-wearable BIA device was only 7 s and could be reduced further, this sensor technology provides a new possibility for a wearable bioelectrical impedance analyzer with more miniature electrodes toward daily obesity management.
